# Cubital Tunnel Revision After Transposition: A Single Center Experience

**DOI:** 10.1016/j.jhsg.2025.100815

**Published:** 2025-08-22

**Authors:** Kathryn S. King, Reed Wulbrecht, Mariel McLaughlin, Victor T. Hung, Jeffrey Stone, Alfred V. Hess, Michael J. Garcia

**Affiliations:** ∗ Hand and Upper Extremity Surgery, Florida Orthopaedic Institute, Tampa, FL; † Department of Plastic and Reconstructive Surgery, Morsani College of Medicine, University of South Florida, Tampa, FL; ‡ Foundation for Orthopaedic Research and Education, Tampa, FL

**Keywords:** Cubital tunnel syndrome, Revision, Subcutaneous, Subfascial, Submuscular transposition

## Abstract

**Purpose:**

The purpose of this study was to assess the rates of revision after cubital tunnel release with transposition among three different transposition techniques in a single institution.

**Methods:**

A retrospective chart review of all cubital tunnel surgeries over a 5-year period was performed via a query of the billing records of three different surgeons who typically perform three different types of transposition. This yielded 937 records. After eliminating records with incomplete clinical information (141 records), a total of 796 records were evaluated, with 540 representing in situ releases and 255 transpositions. The transposition cohort was further evaluated, and 39 records were eliminated as the operation was performed for traumatic or post-traumatic indications, leaving 216 transpositions performed between December 1, 2016, and December 1, 2021.

**Results:**

In the 216 cubital tunnel releases with transposition performed, 82 (38%) were subcutaneous transpositions, 71 (33%) were subfascial transpositions, and 63 (29%) were submuscular transpositions. Twenty of the 216 cubital tunnel releases with transpositions that were performed in this study period represented revision surgeries. Eleven were revisions after an in situ release, and eight were revisions after a transposition. One is unknown as the index operation was performed by an outside physician whose operative note was not available. Of the revision surgeries performed, 10 represented revisions of index cases performed by our institution with six being revisions for an in situ release and four revisions after a transposition. Of those four revisions after a transposition, one was performed for a subcutaneous transposition, two were following subfascial transpositions, and one was following a submuscular transposition. The average time from index operation to revision after a transposition was 16.3 months.

**Conclusions:**

The rate of revision surgery following cubital tunnel release with transposition is quite low, and there do not appear to be major differences in the rate of revision among the different types of surgical transposition, indicating that a true subcutaneous transposition may be adequate.

**Type of study/level of evidence:**

Therapeutic III.

Cubital tunnel syndrome occurs in approximately 30 of every 100,000 people per year in the United States, and over 40% of those people are treated surgically.^1^ Cubital tunnel syndrome is characterized by paresthesia within the ulnar nerve distribution of the hand and ultimately weakness of the intrinsic muscles of the hand. The mainstay of surgical treatment for cubital tunnel syndrome is decompression of the ulnar nerve with or without transposition of the nerve over the medial epicondyle. Multiple studies have supported in situ release without transposition as adequate for primary cubital tunnel releases.^2^^,^^3^ The decision to perform a transposition is typically influenced by presence of subluxation on examination or muscle atrophy; however, the clinical threshold varies among surgeons.^4^

The specific type of transposition performed is surgeon dependent. Some argue that subcutaneous transpositions lead to less postoperative pain and earlier mobilization, whereas advocates for submuscular transposition indicate that the muscle provides a healthier vascular bed with less scarring.^5^^,^^6^ Multiple planes of transposition have been described; however, comparison is frequently made between subcutaneous and submuscular techniques.^7^^,^^8^ Subcutaneous techniques are frequently described with the use of a fascial flap indicating fascial dissection.^8^^,^^9^ Although the nerve is in the subcutaneous plane with this type of transposition, it does require fascial dissection, and the flap could potentially cause a site of compression or scarring. The present study examines the rate of revision surgery after three different types of transposition, including subcutaneous without a fascial flap, subfascial (indicating fascial dissection with flap), and submuscular, at a single institution to determine if there is any true difference. We hypothesized that the rate of revision surgery would not differ among all three techniques.

## Materials and Methods

### Study sample

The billing system of the institution was queried using Current Procedural Terminology code 64718 (neuroplasty and/or transposition; ulnar nerve at elbow) and to identify patients who underwent cubital tunnel release by three different surgeons from a single practice between December 1, 2016, and December 1, 2021. Retrospective chart review was then performed with review of operative and clinical records. Those patients who underwent in situ release were excluded from analysis unless a later transposition was performed. Inclusion criteria included patients over 18 who underwent cubital tunnel release with or without transposition. Exclusion criteria included post-traumatic cubital tunnel syndrome, cubital tunnel release as an adjunct to a trauma case, and patients in whom complete records were not available. International review board approval was obtained for this study.

### Operative procedures

Three different transposition techniques were included: subcutaneous, subfascial, and submuscular. The cubital tunnel releases were performed similarly among all three surgeons by first entering the interval between the medial intermuscular septum and the triceps and then longitudinally dividing the flexor pronator fascia. The flexor carpi ulnaris aponeurosis was divided at the region of impingement as distally as possible, with care to protect the motor branches in this plane. Next, the medial intermuscular septum was carefully dissected, and a portion of the medial intermuscular septum was resected. After release of the ulnar nerve, the elbow was taken through a range of motion, and a clinical decision was made by the operating surgeon regarding the need for transposition. In all transpositions the ulnar nerve was dissected free of the cubital tunnel from proximal to distal.

Subcutaneous transpositions were performed following standard cubital tunnel release. After the release of the ulnar nerve, the flexor pronator muscle was further exposed with elevation of the thick anterior flap, creating a pocket anteriorly for the ulnar nerve, with care of the medial antebrachial cutaneous nerve. Afterwards, the ulnar nerve was transposed anteriorly into the subcutaneous pocket, and the Osbornes ligament and the triceps fascia was closed over the cubital tunnel.

Subfascial transpositions were performed by first exposing and excising the medial intermuscular septum. An oblique lengthening incision was then made in the flexor pronator fascia, and a loose fascial sling was created from the flexor pronator fascia and based proximally. The ulnar nerve was transposed anteriorly into a superficial intramuscular position, and the fascial sling from the flexor pronator mass was placed over the nerve and secured in this plane. The anterior skin flap and subcutaneous fat were secured to the medial epicondylar region as a further guardrail to prevent hypermobility of the ulnar nerve as well as provide an additional healthy bed for the ulnar nerve.

Submuscular transpositions were performed by dissecting to the extent of the flexor pronator fascia as distally as possible with care to protect the distal cutaneous nerve branches. The flexor carpi ulnaris aponeurosis was divided distally as possible. Then, a lengthening incision was made after exposing the flexor pronator muscle fascia. Subsequently, dissection was carried down through the musculature of the flexor pronator mass. The ulnar nerve was then transposed anteriorly and placed within the flexor pronator muscle group. The fascia was approximated in a lengthening fashion for the flexor pronator group to reach the appropriate length and then closed over top of the transposed ulnar nerve.

### Primary outcome measure and sample size analysis

The primary outcome measure of this study is the rate of revision surgery for each cubital tunnel release technique. Based on previous studies, the rate of cubital tunnel revision surgery for ulnar nerve transpositions was found to be 11.1%.^10^ At our institution, preliminary studies found that the average rate of revision surgery for elective ulnar nerve transpositions was approximately 2.5%. Therefore, with an alpha of 0.05, a calculated sample size of at least 42 subjects per group was required to power the study at 0.80, where a difference of more than 5% in favor of the standard group will be excluded.

### Statistical analysis

Averages and ranges were reported for continuous variables and percentages were reported for categorical variables. Comparisons between categorical variables were performed using chi-square or Fisher’s exact test, and between continuous variables were performed using independent student t-test and paired t-test, where appropriate. All analyses were performed using SPSS software (version 25; IBM) with statistical significance set at *P* <0.05.

## Results

**Figure 1:**
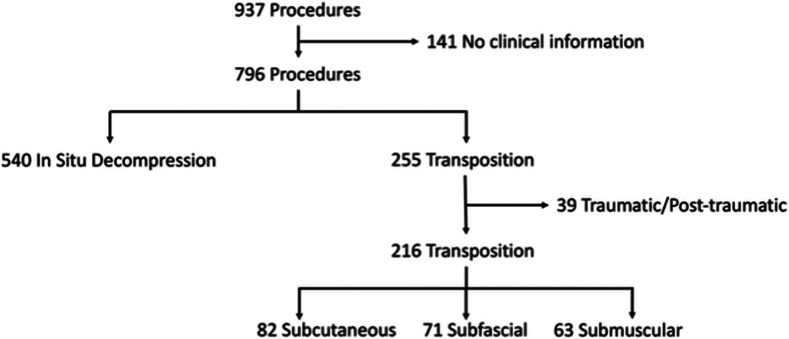
Study inclusion and exclusion flowchart.

A total of 937 cubital tunnel releases were performed by three surgeons between December 1, 2016, and December 1, 2022. After excluding cases for lack of appropriate clinical documentation (141 records), we had a total of 540 in situ releases (68%) and 255 transpositions (32%) during this study period. The 255 transpositions were further evaluated, and 39 procedures were performed for traumatic of post-traumatic reasons and were discarded leaving 216 transpositions for review (Fig. 1).

**Figure 2:**
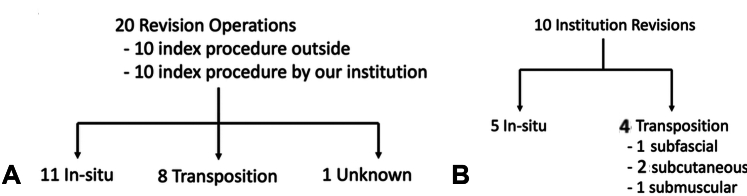
Revision procedures. **A** Overall revisions. In total, 50% of revision surgeries were performed on patients whose index operation was performed at another institution. The majority of revisions were performed for in situ releases. **B** Institution revisions. In total, 50% of revisions were performed on patients who had index operations at our institution. In addition, 60% of revisions were performed after in situ release, and 40% were performed after a transposition.

Of the 216 transpositions performed in this timeframe, 82 patients (38%) received a subcutaneous transposition, 71 (32.3%) a subfascial transposition, and 63 (29%) a submuscular transposition. Of these 216 transpositions performed, 20 (9%) were revision surgeries. Of these 20 procedures, 10 had the index operation performed at our institute, and 10 were performed elsewhere. Of the 10 revisions where the index operation had been performed at our institute, five were revisions of an in situ release, and five were revisions of a transposition. The average time from index operation to revision for these 10 patients was 16.3 months (2–32 months). Of the five revised after a transposition, three were revised after a subfascial transposition, one after a submuscular transposition, and one after a subcutaneous transposition (Fig. 2A and B).

The total number of revision operations in the five-year time period study was 20, representing 2.5% of all cubital tunnel operations performed. Of those revisions, eight (40% of revisions, 1% of all cubital tunnel releases) were performed after a transposition, and 11 (55% of revisions, 1.4% of all cubital tunnel releases) were performed after an in situ release. In one patient, the index operation was performed by an outside surgeon, and we were unable to determine if the nerve was left in situ or transposed. Of the patients who underwent a revision operation after an index procedure with our group, six (60%) of the revisions were for in situ releases, and four (40%) were following a transposition. In looking more critically at revisions following a transposition, one patients had undergone a subfascial transposition, two patients had undergone a subcutaneous transposition (which represented a revision from an anterior transposition of an unclear subtype performed by an outside surgeon), and one had undergone a submuscular transposition. There were no significant differences in the rate of revision for all three types of ulnar nerve transpositions (*P* > .597). In situ releases were most often revised to a submuscular plane (8/11) (Table 1).

**Table 1:** Case Details of Revision Tranposition Patients

Patient	Index Operation Location	Index Operation	Transposition Plane Revised To	Further Revisions	Time for Index Operation to Revision (mo)	Follow-up (mo)
1	OSH	In situ	Subcutaneous	No	24	1
2	OSH	In situ	Subfascial	No	11	12
3	OSH	In situ	Submuscular	No	124	14
4	OSH	In situ	Submuscular	No	10	1
5	OSH	N/A	Submuscular	No	N/A	6
6	OSH	Endoscopic/in situ	Submuscular	No	22	2
7	FOI	In situ	Subcutaneous	No	2	2
8	FOI	In situ	Submuscular	No	19	2
9	FOI	In situ	Submuscular	No	32	4
10	FOI	In situ	Submuscular	No	25	8
11	FOI	In situ	Submuscular	No	12	2
12	FOI	In situ	Submuscular	No	30	1
13	OSH	Unknown type of transposition	Subcutaneous	Yes, neurolysis and neuroma excision	15	39
14	OSH	Submuscular	Submuscular	No	84	3
15	FOI	Subfascial	Submuscular	No	14	0
16	OSH	Unknown type of transposition	Subfascial	No	36	9
17	FOI	Subfascial	Subcutaneous	No	18	7
18	OSH	Submuscular	Subcutaneous	No	5	5
19	FOI	Subfascial	Subcutaneous	No	7	2
20	FOI	Subfascial	Subfascial	No	13	14

FOI, Florida Orthopaedic Institute; OSH, Outside Surgical Hospital; N/A, not available

Demographics data of the patients were similar among the different types of transposition (Table 2). Demographics for the patients requiring revision were also examined but the number of revisions in total is quite low so making meaningful assessments of risk factors for revision from this data set is not possible (Table 3).

**Table 2:** Ulnar Nerve Transposition Patient Demographics

	Total	Transposition Type		
		Subcutaneous	Subfascial	Submuscular
Total, n (%)	216 (100)	82 (38)	71 (33)	63 (29)
Age				
Average (range)	53 (19–87)	55 (19–87)	54 (20–84)	51 (23–79)
**Comorbidities, n (%)**				
Cervical spine disease	50 (23)	12 (15)	13 (18)	25 (40)
Diabetes mellitus	27 (12)	14 (17)	6 (8)	7 (11)
Tobacco	23 (11)	8 (10)	5 (7)	10 (16)
Working	123 (57)	47 (57)	34 (48)	42 (67)
Follow-up (mo)	8.85	7.68	10.2	8.87

**Table 3:** Revision Transposition Patient Demographics

	Total	Unknown	In Situ	Transposition Type		
				Subcutaneous	Subfascial	Submuscular
Total, n (%)	20	1	11	1	5	2
Age						
Average (range)	51 (29–80)	62	54 (37–80)	57	45 (31–52)	42 (29–55)
**Comorbidities,n (%)**						
Cervical spine disease	5 (25)	1 (100)	2 (18)	1 (100)	1 (25)	0
Diabetes mellitus	2 (10)	0	1 (9)	0	1 (25)	0
Tobacco	3 (15)	0	2 (18)	0	1 (25)	0
Working	11 (55)	1 (100)	6 (55)	0	2 (50)	2 (100)
Follow-up (mo)	5.2	6	4.83	3	5.75	7

## Discussion

Although prior studies have investigated the revision rates of cubital tunnel releases after in situ decompression versus anterior transposition, few look specifically at revision rates after different types of transpositions. There are three main types of transposition following a cubital tunnel release, and they involve a varied degree of dissection of the surrounding tissue. In all transpositions, the nerve is mobilized and moved out of the cubital tunnel anterior to the medial epicondyle. The variety in transposition techniques comes from the degree of dissection of the surrounding tissue. In this study, subcutaneous transpositions were defined as those in which no additional dissection was performed other than mobilization of the nerve and the tunnel was closed down by suturing the fascia of the medial epicondyle to Osbornes and triceps fascia. Subfascial transpositions were defined as any transposition that required dissection of the fascia off the flexor pronator mass, such as the creation of a fascial flap/bumper. Submuscular transpositions were defined as those that involved dissection into the musculature of the flexor pronator mass itself. Here, we present retrospective review of 796 cubital tunnel releases over a 5-year period with an overall 2.5% revision rate (20 revisions out of 796 cubital tunnel releases). We found no significant differences in the rate of revision between all three transposition techniques.

Previous studies have looked at differences in complication and revision rates among different transposition techniques and have had differing findings. A 2015 meta-analysis by Liu et al^7^ found the quality of evidence in studies attempting to elucidate the ideal anterior transposition technique to be poor overall; however, they suggested that subcutaneous and submuscular transposition techniques have similar efficacy. A 2014 retrospective multicenter review found higher recurrence rates with submuscular transposition versus subcutaneous and no difference in complication rates though they admittedly had a very low number of recurrences in general.^8^ In our study, we demonstrate a relatively equal distribution of the different transposition techniques, including 37% for subcutaneous, 32% for subfascial, and 31% for submuscular, with revision rates being higher among the subfascial group 2 of 71 (2.8%) versus 1 of 63 (1.6%) in the submuscular group and 1 of 81 (1.2%) in the subcutaneous transposition group in this study population. Although the numbers are quite low, consistent with most of the literature, we demonstrate higher rates of secondary surgery in patients who underwent transposition requiring dissection of either the fascia or muscle as compared to transposition into the subcutaneous plane alone without additional fascial or muscular dissection. This begs the question: do we need to perform more dissection in primary cubital tunnel releases to place the nerve in the submuscular or subfascial plane if the rate of secondary surgery is similar among all technique?

The overall revision rate for cubital tunnel releases are quite low. However, the literature is varied with some reports citing a 2.8% revision rate, similar to our study, and others up to 18%.^10^, ^11^, ^12^ The reported rate of revision after in situ decompression versus anterior transposition is also varied with some reports demonstrating no major difference in revision rate between in situ release and transposition^10^ and others citing a secondary surgery rate of 11.1% for transpositions with only a 2.5% rate for in situ decompression.^10^ Here, we present a relatively low revision rate of 2.5% overall broken down into a rate of 1.4% revision after an index in situ release (11 out of 796 cubital tunnel releases) and 1% revision after an index transposition (8 out of 796 cubital tunnel releases). It should be noted that we excluded cases performed for a trauma (ie, mobilization and transposition of the nerve in treatment of a distal humerus fracture) as well as post-traumatic cases (ie, mobilization and transposition of the nerve associated with contracture release or removal of hardware post-traumatically). This likely introduced some selection bias as other studies have demonstrated an increased need for revision surgery for cubital tunnel syndrome associated with trauma.^10^ The indications for our cubital tunnel revision surgeries were prolonged persistent or worsening symptoms after the primary cubital tunnel release, with recurrence confirmed with a repeat nerve conduction or EMG study. Our threshold for revision was based primarily on patient symptoms and nerve conduction and/or EMG studies regardless of the presence of ulnar nerve subluxation after the primary surgery.

The limitations of this study are inherent to its retrospective design. We likely underestimated the rate of revision as patients may have followed up with other providers for recurrent symptoms. Additionally, our study focused on the patient population for elective cubital tunnel releases and excluded cases associated with a history of trauma, which is a source of selection bias. In this study, 50% of the revision surgeries were performed on patients whose primary operation was performed by a physician outside our group. When patients fail to respond to an intervention, or develop recurrent symptoms, they may be more likely to seek care elsewhere. We did find that our average follow-up time was 8.7 months. It should be noted, however, that the average time from index operation and revision surgery in those who required a secondary intervention was 26 months overall (range 2–124 months) and 16 months (range 2–32 months) for those patients whose index and revision surgery were performed by our group. This is consistent with what we know about recovery rate of nerves after decompression. The nerve recovers at a rate of 1 mm/day on average; thus, we often counsel patients that it may take 1 year to 18 months for full recovery of the ulnar nerve from cubital tunnel decompression. If we take this into consideration, we would ideally be following up with all cubital tunnel release patients at 1 year and 18 months after intervention for adequate assessment of their recovery. We must also consider the difference between incomplete resolution of symptoms versus recurrence of symptoms. This can be harder to elucidate as it requires subjective assessment from the patient. Electrodiagnostic studies can certainly be helpful here for an objective measure and have been used previously.^7^ Another limitation to our study was not including the electrodiagnostic results before surgery or assessing their use after surgery in those patients requiring a revision. Further studies with a prospective design that includes diagnostic work-up are warranted.

Overall, this single institution retrospective review demonstrates low overall revision rates following cubital tunnel with transposition and the safety and efficacy of subcutaneous transposition over subfascial and submuscular transpositions.

## Conflicts of Interest

No benefits in any form have been received or will be received related directly to this article.
